# Nadir PSA is a strong predictor of treatment outcome in intermediate and high risk localized prostate cancer patients treated by definitive external beam radiotherapy and androgen deprivation

**DOI:** 10.1186/s13014-017-0884-y

**Published:** 2017-09-07

**Authors:** Fady B. Geara, Muhammad Bulbul, Raja B. Khauli, Therese Y. Andraos, Mirna Abboud, Abdelatif Al Mousa, Nasim Sarhan, Ahmed Salem, Hamza Ghatasheh, Anoud Alnsour, Zeina Ayoub, Ibrahim Abu Gheida, Maya Charafeddine, Mohammed Shahait, Ali Shamseddine, Rami Abu Gheida, Jamal Khader

**Affiliations:** 1 Department of Radiation Oncology, The Naef K. Basile Cancer Institute at the American University of Beirut Medical Center, Bliss Street, Riad El Solh, Beirut, 11072030 Lebanon; 2 Division of Urology, the American University of Beirut Medical Center, Beirut, Lebanon; 3 Department of Radiation Oncology, King Hussein Cancer Center, Amman, Jordan; 4 Division of Medical Oncology, The Naef K. Basile Cancer Institute at the American University of Beirut Medical Center, Beirut, Lebanon

**Keywords:** Prostate cancer, External beam radiation therapy, Androgen deprivation, Nadir PSA

## Abstract

**Background:**

The aim of this study is to investigate the effect of tumor characteristics and parameters of treatment response in predicting biochemical disease-free survival (BFS) for patients with intermediate or high risk prostate cancer treated by combined definitive external beam radiation therapy (EBRT) and androgen deprivation therapy (ADT).

**Methods:**

Between June 1995 and January 2015, 375 patients with localized prostate cancer and a National Comprehensive Cancer Network (NCCN) intermediate or high risk categories were treated by definitive EBRT and ADT. Median duration of androgen blockade was 10 months (range: 3–36 months); Median radiation dose was 72 Gy (Range: 70–78 Gy). Median follow-up time was 5.8 years (range: 0.8–16.39 years). The main study endpoint was biochemical disease free survival (BFS).

**Results:**

Forty seven patients (12.5%) developed biochemical recurrence (BCR) during the observation period. Monovariate analysis identified baseline PSA (bPSA) (*p* = 0.024), T-stage (*p* = 0.001), Gleason’s score (GS) (*p* = 0.042), radiation dose (*p* = 0.045), PSA pre-radiation therapy (*p* = 0.048), and nadir PSA (nPSA), (*p* < 0.001) as significant variables affecting BCR. The receiver operating characteristic (ROC) curve identified a nPSA of 0.06 ng/ml as optimal cut-off value significantly predicting the patients’ risk of BCR (*p* < 0.001). Multivariate cox regression analysis revealed T-stage, GS, and nPSA as independent variable affecting BFS, while bPSA, age, and radiation dose were not.

**Conclusion:**

Nadir PSA at 0.06 is a strong independent predictor of BFS in patients with intermediate or high risk prostate cancer treated by definitive EBRT and ADT.

## Introduction

Intermediate and high risk localized prostate cancers (PrCa) are effectively treated by definitive external beam radiation therapy (EBRT) in combination with androgen deprivation therapy (ADT). Several large intergroup phase III trials, have demonstrated that the combination of ADT and EBRT lead to a significant improvement in prostate cancer specific mortality (PCSM), distant metastasis, and biochemical recurrence in those patients [1–3]. Prostate-specific antigen (PSA) is an important marker in screening and monitoring prostate cancer patients. It is also a well-established prognostic factor in determining the risk of relapse. During the treatment process, baseline PSA (bPSA) typically starts declining indicative of a good response. The lowest PSA levels or nadir (nPSA) is ultimately reached after several weeks or months. The absolute value of nPSA is typically lower and is reached much faster when ADT is added to EBRT [4].

Several studies have analyzed the prognostic value of PSA measurements taken at variable time intervals during and after the course of treatment. These measurements included PSA halving time, PSA post ADT and pre-radiation therapy, PSA immediately post radiation therapy, and nPSA [5–7]. Studies that used nPSA as endpoint have demonstrated that this parameter is an important determinant of outcome that separates patients with good or bad prognosis (below and above the nadir, respectively). However, there is no consensus on the absolute value of nPSA as this parameter’s cutoff or assessment method varied widely between studies [4, 8–12]. A probable cause for this variation, could be the composition of the studied patient populations which very often contained a mix of patients treated either by radiation alone or a combination of radiation with or without ADT which could influence the spectrum of PSA response.

The aim of this study is to investigate the effect of nPSA along with other known tumor characteristics such as T-stage, Gleason’s score, and bPSA in predicting biochemical disease-free survival (BFS) in intermediate and high risk prostate cancer patients, all being treated with a well-defined protocol of combined EBRT and ADT.

## Materials and methods

This study included two patient populations treated at two institutions in the Middle East: the Naef Basile Cancer Institute (NBCI) at The American University of Beirut, Lebanon and the King Hussein Cancer Center (KHCC) Amman, Jordan. Both institutions are leading academic tertiary referral center for cancer diagnosis and management and have many research and academic programs in common. Between January 1998 and July 2015, a total of 509 Pca patients were seen at both institutions for treatment by definitive radiation therapy. Of those, 375 patients (213 from NBCI and 162 from KHCC) had a National Comprehensive Cancer Network (NCCN) intermediate or high risk category and were treated with EBRT and concomitant ADT and were retained for this study. The study was approved by the Institutional review boards of both institutions.

The patients’ medical records were accessed through the hospital and clinic charts and/or electronic medical records. Data collection included demographic data, baseline tumor characteristics, radiation and hormonal therapy data, toxicity, tumor response parameters, and data on disease recurrence. Patients were stratified into risk groups according to the NCCN criteria for prostate cancer.

Androgen deprivation therapy (ADT) included Luteinizing-Hormone-Releasing Hormone agonist (LHRH) alone or in combination with anti-androgen therapy, for a total duration ranging from 3 to 36 months. A total of 159 patients (42%) received ADT for less than 6 months, 127 patients (34%) between 6 and 24 months, and 75 (20%) patients had more than 24 months. The reason patients received less than 6 months is predominantly because of patient compliance, and more than 24 months is due to physician preference. For 14 patients (4%) the duration of ADT could not be retrieved. Radiation therapy was delivered by 3D–Conformal Radiation Therapy (3D–CRT) or Intensity-Modulated Radiation Therapy (IMRT) with doses ranging from 70 to 78 Gy. The majority of patients (327 patients; 87%) received a dose equal or higher than 72 Gy, whereas 44 patients (12%) received a dose lower than 72 Gy. Conformal 3DCRT was used for 213 patients (57%) and IMRT for 160 patients (43%).

PSA levels after ADT and EBRT were typically obtained every 4 months the first 2 years and every 6 months thereafter. These values were recorded, and the lowest PSA value attained was considered as the nadir PSA. Median Time to nadir was defined as time from end of RT till nadir PSA is achieved. Biochemical recurrence (BCR) was defined as “nPSA +2 ng/ml” based on the Phoenix definition [13]. Time to biochemical recurrence was calculated from the time of end of RT till time of recurrence. Median follow-up time from the end of RT for all the patients was 5.8 years (0.8–16.39), [14]. Two hundred seventy patients (73%) had a minimum follow-up of 3 years.

Biochemical disease free survival rates were estimated using the Kaplan-Meier method and the various groups were compared using the log-rank test. To identify the optimal cutoff for the nadir PSA level, the receiver operating characteristic (ROC) curve was plotted. Equal weight to sensitivity and specificity was given to select the optimal cutoff for patients who had a higher risk of biochemical recurrence. Cox survival analysis was employed for the univariate and multivariate analyses to examine prognostic factors for biochemical recurrence. The included variables were age, bPSA, T-stage, Gleason’s score, nPSA, radiation dose, ADT duration, PSA pre-RT, and time to nadir. Using the backward elimination, the hazard ratios (HRs) and 95% confidence intervals (CIs) were calculated for variables that remained significant in the model. All *p* values are 2-sided; a value of *p* < 0.05 was considered significant. All statistical analysis was performed using the SPSS v.23.0 statistical package.

## Results

### Patient characteristics

Table 1 shows patient demographics and tumor characteristics. Median age was 71 years (range: 51–92 yrs.). The majority of patients had stage T1-T2 disease (261 patients; 70%), and 46% of them had Gleason’s score of 7. There was more high risk than intermediate risk patients (62% vs 38% respectively). PSA on presentation was almost equally divided between the main risk categories (<10: 32%; 10–20: 35%; > 20:32%).

**Table 1:** Patient and treatment characteristics

Characteristic	Number	Percent
Total	375	100
Median age (yr)	71 (51–92)	
≤ 70 yr	185	49.3
> 70 yr	190	50.7
T stage		
T1-T2	261	69.6
T3-T4	103	27.5
Unknown	11	2.9
NCCN risk group		
Intermediate	143	38.1
High	232	61.9
Gleason Score		
< 8	241	64.3
8–10	133	35.5
Unknown	1	0.3
PSA on presentation		
≤ 20 ng/ml	253	67.5
> 20 ng/ml	118	31.5
Unknown	4	1
Median ADT duration (months)	10 (3–36)	–
Median Radiation Dose (Gy)	72 (70–78)	–
< 72	44	11.7
≥ 72	327	87.2
Unknown	4	1.1

*NCCN* National Comprehensive Cancer Network, *ADT* androgen deprivation therapy, *PSA* Prostate-Specific Antigen

### Patient outcome

**Fig. 1:**
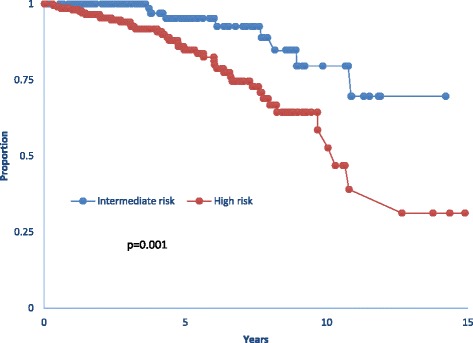
Kaplan Meier survival curve of biochemical free survival for intermediate and high risk patients. Median survival for high risk patients was 10.3 yrs. Median survival was unreached. For intermediate risk patients

Forty seven patients of the entire group developed biochemical relapse for an estimated 5 and 10-years BFS rates of 88.6 and 66.4%, respectively. Intermediate risk patients had a higher 5 and 10-years BFS rates (95.3 and 79.6%, respectively) compared to high risk patients (84.7 and 58.4%, respectively). This difference between the two risk groups is statistically significant (*p* = 0.001); Fig. 1.

### Baseline PSA and nadir PSA

Intermediate risk patients had a significantly lower PSA on presentation (10.99 ng/ml) compared to high risk patients (30.72 ng/ml); (*p* < 0.001). The receiver operating characteristics (ROC) curve revealed an optimal cut-off value for bPSA of 14.55 ng/mL, above which the risk of biochemical recurrence (BCR) increased significantly. BCR developed in 10.2% of patients with a bPSA <14.55 ng/mL compared to 18.4% of patients with bPSA ≥ 14.55 ng/mL (*p* = 0.03). In addition, patients with bPSA < 14.55 ng/mL achieved a lower absolute PSA levels on neoadjuvant ADT alone as noted from pre radiation PSA (preRT PSA) values, (*p* < 0.001), and a lower Nadir PSA (*p* = 0.019) compared to those with bPSA above 14.55 ng/ml. Median nPSA was 0.02 ng/ml. Patients who ultimately developed biochemical recurrence had a higher mean nPSA post RT compared to those patients who had no recurrence (0.33 ng/ml vs. 0.07 ng/ml; *p* < 0.001). The ROC curve revealed an optimal cut-off value for nPSA of 0.06 ng/ml, with a significant increase in the risk of biochemical recurrence in patients having a nPSA ≥ 0.06 ng/ml. Among patients who had a nPSA < 0.06 ng/mL (*n* = 234 patients), only 15 patients (6.4%) developed biochemical recurrence, while 20 out of 78 patients (25.6%) with nPSA ≥ 0.06 ng/mL developed biochemical relapse (*p* < 0.001). Median Time to nadir was 6 months (range = 0.93–108 months). In our data set, there was no significant correlation between time to nadir and the risk of biochemical recurrence.

### Univariate and multivariate analyses

**Fig. 2:**
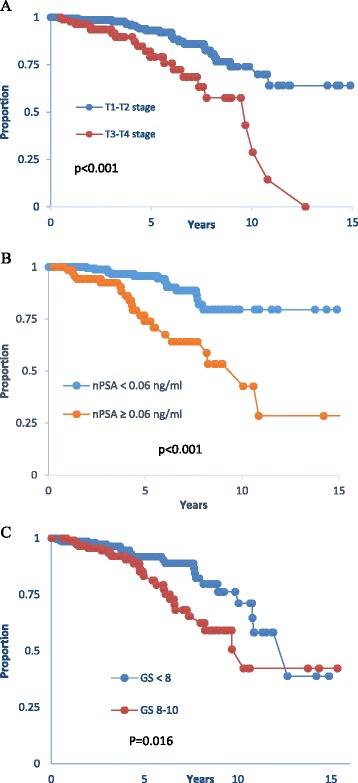
**a** Kaplan Meier survival curve of biochemical free survival rates for T1-T2 versus T3-T4 stage. **b** Kaplan Meier survival curve of biochemical free survival as a function of nadir PSA (nPSA) with the cutoff of 0.06 ng/ml. **c** Kaplan Meier survival curve of biochemical free survival rates for patients with Gleason’s score (GS) < 8 and those with GS 8–10

**Fig. 3:**
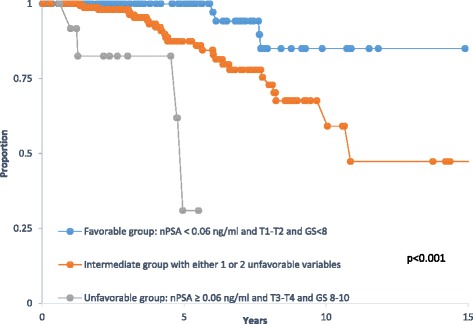
Kaplan Meier survival curve of biochemical free survival rates by risk categories as a function of nadir PSA (nPSA), T-stage, and Gleason's score (GS)

Using BFS as endpoint, the following variables were used for univariate analysis: GS, T stage, bPSA, nPSA, pre-RT PSA, radiation dose (RD), age, ADT duration, and time to nadir. GS (*p* = 0.042), T stage (*p* = 0.001), bPSA (*p* = 0.024), nPSA (*p* < 0.001), pre-RT PSA (*p* = 0.048), radiation dose (*p* = 0.045) were significantly associated with BFS, while age, ADT duration, and time to nadir were not (Table 2). Significant variables in the univariate model were then studied in a multivariate cox regression analysis. The model retained T-stage, nPSA, and GS as the only independent variables to significantly affect BFS (Table 3). Patients with stage T1-T2 disease had a BFS at 5- and 10- year of 93 and 74% respectively compared to 79 and 43% for those with stage T3-T4 stage disease (Fig. 2a). Also patients who had a nPSA below 0.06 ng/ml had a 5- and 10-year BFS of 96 and 80%, respectively compared to 74 and 53% for those who had a nadir above the cut-off value of 0.06 ng/ml (Fig 2b). For GS, the best cut-off grouping was found for grades lower than 8 (< 8) versus GS 8–10; patients with GS < 8 had a 10-year BFS of 74% compared to 52% for those with GS 8–10 (Fig 2c). Using these three parameters, we then grouped patients into 3 categories with regard to their risk of BFS: A favorable group with a combination of nPSA < 0.06 ng/ml, T1-T2 stage disease, and GS < 8; an unfavorable group with nPSA ≥ 0.06 ng/ml, T3-T4 stage disease, and GS 8–10; and an intermediate group containing one unfavorable variable. Five-year BFS rates were 100% for the favorable group, 87% for the intermediate, and 31% for the unfavorable group (*p* < 0.001); (Fig 3).

**Table 2:** Univariate analysis of factors affecting biochemical free survival

Factor	HR (95% CI)	*p*-value
GS	1.299 (1.009–1.672)	0.042
< 8	1 (reference)	–
8–10	2.007 (1.129–3.569)	0.018
bPSA (ng/ml)	1.009 (1.001–1.017)	0.024
≤ 20 ng/ml	1 (reference)	–
> 20 ng/ml	1.767 (0.970–3.218)	0.063
T-stage	2.226 (1.410–3.515)	0.001
T1-T2	1 (reference)	–
T3-T4	3.079 (1.691–5.608)	<0.001
nPSA (ng/ml)	2.667 (1.79–3.973)	<0.001
< 0.06	1 (reference)	–
≥ 0.06	3.887 (1.988–7.599)	<0.001
RT dose (Gy)	0.837 (0.703–0.996)	0.045
PSA pre-RT (ng/ml)	1.056 (1.0–1.114)	0.048
ADT duration (months)	0.996 (0.970–1.023)	0.784
Age (years)	0.968 (0.926–1.012)	0.148
Time to Nadir (months)	0.999 (0.969–1.031)	0.965

**Table 3:** Multivariate analysis of factors affecting biochemical free survival

Factor	HR (95% CI)	*p*-value
T-stage	1.84 (1.073–3.155)	0.027
T1-T2	1 (reference)	–
T3-T4	2.886 (1.408–5.916)	0.004
GS	1.219 (0.887–1.677)	0.222
< 8	1 (reference)	–
8–10	2.449 (1.214–4.940)	0.012
nPSA (ng/ml)	2.306 (1.503–3.537)	<0.001
< 0.06	1 (reference)	–
≥ 0.06	4.409 (2.180–8.916)	<0.001
bPSA (ng/ml)	1.003 (0.992–1.014)	0.649
≤ 20 ng/ml	1 (reference)	–
> 20 ng/ml	1.641 (0.765–3.520)	0.203
RT dose (Gy)	0.916 (0.738–1.137)	0.425

## Discussion

This study included 375 patients with intermediate or high risk prostate cancers, all treated by combined radiation and androgen deprivation therapy. Our main findings were that nadir PSA, along with T- stage, and Gleason’s score are the most powerful independent prognostic factors predicting the risk of biochemical recurrence. While T-stage, and GS are well-known and established prognostic factor, which represent tumor characteristics that are known at diagnosis, nPSA is a treatment outcome variable and a less established prognosticator, that is only known after treatment is completed. The optimal cutoff value for nadir PSA was 0.06 ng/ml, above which patients were at an increased risk of BCR. Like many independent variables, when used in combination, patients were better segregated by outcome: favorable with 100% BFS at 5 years, intermediate with 87%, and unfavorable with 31% biochemical control at the same time point.

The present study combined two patient populations from two different institutions: NBCI and KHCC. The analysis was first carried on each population separately and the results were found to be very similar with regard to patient characteristics, treatment parameters, and outcome analysis for both univariate and multivariate analyses. The nPSA cutoff value found in our population at the NBCI was 0.056 ng/ml. This nPSAvalue was then tested on the KHCC patient population and was found to be equally significant for BCR, with patients from this group who had a nPSA above 0.056 having a higher rate of biochemical failure than those who had a nPSA below 0.056 (14.7% vs 3.1%; respectively; *p* = 0.009). When we combined both patient populations, the ROC curve fit identified a slightly higher cutoff value for nPSA, 0.06 ng/ml (instead of 0.056), and this value was retained for the remainder of the analysis.

**Fig. 4:**
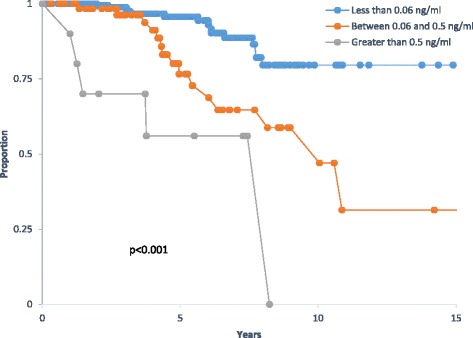
Kaplan Meier survival curve of biochemical free survival by nadir PSA (nPSA) using two different cutoffs 0.5 and 0.06 ng/ml; (*p* < 0.001)

There are several studies in the literature that examined nPSA as a determinant of outcome in prostate cancer patients treated by definitive radiotherapy with or without ADT [4, 8–12, 15, 16]. There are many differences between our study presented here and those studies. These are related to treatment protocols (with or without androgen deprivation) and nPSA cutoff values. The majority of the studies included patients treated either by radiation alone or a mix of radiation alone with or without ADT. Cutoff values for nPSA varied between 0.1 and 0.7 ng/ml for studies that used nPSA as a continuous or dichotomized variable [4, 11, 12, 15], and between 1.5 and 2 ng/ml for studies that used a PSA value a fixed time point like 1 or 2 years [8–10]. Some other studies have focused on PSA halving times during the neoadjuvant period before the start of RT [5, 6, 17]. As expected, the addition of androgen blockade to radiation produces more PSA suppression and the studies that included patients treated with ADT have reported lower cutoff values for nPSA [4, 11]. Tseng and colleagues have looked at nPSA in a mixed population treated by RT with or without ADT. Those who were treated with combined therapy had a lower nPSA cutoff (0.1 ng/ml) compared to those treated by RT alone (0.7 ng/ml) [4]. Similar findings were observed by d’Amico and collaborators in a study combining data from two randomized studies from the USA and Australia [11]. In this particular study, the cutoff of 0.5 ng/ml was found to be a good separator between good and poor prognosis patients with regard to prostate cancer specific mortality (PCSM). Moreover, in that study, the authors were able to show that the attaining of the nPSA abolished the benefit of concurrent ADT in that patients who reached a nPSA of 0.5 ng/ml or lower fared very well regardless if they received concurrent ADT or not. We examined this cutoff value (of 0.5 ng/ml) in our dataset and compared it to our cutoff that we determined using the ROC fit (0.06 ng/ml). Figure 4 shows that both cutoff values (0.5 and 0.06) are predictive for BCF in our data. However, the cutoff of 0.06 provided additional prognostic segregation, in that patients who had a nPSA between 0.06 and 0.5 had an intermediate outcome between those with nPSA above 0.5 (unfavorable), and those with nPSA below 0.06 (favorable). This indicates that the cutoff found in our dataset has additional prognostic value and may represent a more powerful predictor at least for the endpoint examined in our study, which is BCF (Table 4). It is of interest to note that Foo and colleagues, who studied a population of predominantly high risk patients treated by combined ADT and radiation therapy, have also found a cutoff value of nPSA similar to ours (0.06 ng/ml) which was highly predictive of BF and prostate cancer specific mortality [5].

**Table 4:** Nadir PSA subgroups and their corresponding 5 and 10-years biochemical free survival

Subcategories	5-years BFS rate (%)	10-years BFS rate (%)
nPSA < 0.06	95.7	79.6
0.06 < nPSA < 0.5	76.6	58.8
nPSA > 0.5	56	0

Other prognostic factors like baseline PSA, pre-radiation PSA, and radiation dose were examined in our study and they were found all significant in univariate analysis (Table 2). However, none of these factors was retained in the multivariate analysis, when we used the usual stratification by risk category, and the only variables retained were nPSA, GS, and T-stage. It is important to note that time to nadir PSA (tnPSA) was not a significant prognostic factor in both univariate and multivariate analyses, whether it was taken as a continuous or dichotomized variable around the median. It was surprising to see that baseline PSA was not retained by the multivariate analysis as an independent prognostic factor. This is a classic and well established prognostic factor for localized prostate cancer and is a determinant, along with T-stage and GS for the widely used risk categorization of the NCCN [18]. In studies comparable to ours that included nPSA in their analyses, it is not uncommon to see that baseline PSA does not come out as a significant independent variable of outcome [4, 6, 10, 12, 15]. This may indicate that the prognostic impact of this baseline tumor characteristic is outplayed by treatment response which is represented by nPSA.

Our results also show that radiation dose and preRT PSA value are important factors in determining BCF by univariate analysis. Our median radiation dose was 72 Gy and those patients who received a dose ≥ 72 Gy fared better. The notion that radiation dose escalation remains effective when patients are treated by combined RT and ADT has been debated for years. However, recent data confirmed the added benefit of high dose radiation in prostate cancer patients receiving ADT [19–21]. Our results on the dose effect are consistent with these data and suggest keeping high radiation doses for patients receiving ADT. Regarding preRT PSA, several studies have demonstrated the predictive value of this parameter. Large single institution and intergroup database have been retrospectively queried and demonstrated that a preRT PSA below the range of 0.3–0.5 ng/ml is predictive of a good outcome [5, 17, 22–24]. In our study, preRT PSA was recorded in 294 out of the 375 patients; 133 of them (45%) reached a preRT PSA ≤ 0.5 ng/mL and achieved a 5-years BFS rate of 90% compared to 85% for those who had a preRT PSA > 0.5 ng/ml (*p* = 0.04). Similar results were obtained when using a PreRT PSA cutoff of 0.3 ng/ml (p = 0.04). Unfortunately, because of missing data for 81 patients, this parameter could not be used in the multivariate analysis.

## Conclusion

In conclusion, our study here has examined the effect of nPSA on biochemical failure for patients with NCCN intermediate and high risk prostate cancer treated by combined androgen ablation and definitive radiation therapy. Along with T-stage, and Gleason’s score, nadir PSA of 0.06 ng/ml was found to be a strong independent predictor of BCF. All three parameters could be used to improve our prognostic classification for those patients and might help initiate early and more individualized therapy, such as second line hormonal ablation or chemotherapy, when there is a high predicted risk of recurrence.
